# Liquid Biopsies in HNSCC: Current Landscape and Emerging Opportunities in the Era of HPV Stratification

**DOI:** 10.3390/ijms27062847

**Published:** 2026-03-20

**Authors:** Akshaya Poonepalle, Jianqiang Yang, Nabil F. Saba, Yang Liu, Yong Teng

**Affiliations:** 1 Wallace H. Coulter Department of Biomedical Engineering, Georgia Institute of Technology and Emory University, Atlanta, GA 30322, USA; apoonepalle6@gatech.edu; 2 Department of Hematology and Medical Oncology, Winship Cancer Institute, Emory University, Atlanta, GA 30322, USA; jqyang100@hotmail.com (J.Y.);; 3 School of Chemical, Materials and Biomedical Engineering, University of Georgia, Athens, GA 30602, USA; liuy@uga.edu

**Keywords:** liquid biopsies, HNSCC, HPV, circulating analytes, precision oncology

## Abstract

Head and neck squamous cell carcinoma (HNSCC) is biologically and clinically dichotomous according to HPV status, a distinction that fundamentally dictates the design, implementation, and interpretation of liquid biopsy strategies. Conventional anatomical imaging lacks sufficient sensitivity for minimal residual disease (MRD) detection, contributing significantly to treatment failure and suboptimal clinical outcomes. This review provides a critical, evidence-based synthesis of the three principal circulating analytes, circulating tumor DNA (ctDNA), exosomes, and circulating tumor cells (CTCs), and their evolving roles in real-time, non-invasive molecular monitoring. Critically, the clinical readiness of these analytes differs substantially: while ctDNA, particularly HPV-related ctDNA, is approaching clinical validation for MRD detection and recurrence surveillance in HPV-positive HNSCC, exosomes and CTCs remain investigational tools hindered by ongoing technical challenges including lack of standardized assays, limited reproducibility across platforms, and insufficient prospective validation. We review how the presence of a clonal, virally derived DNA target in HPV-positive HNSCC contrasts with the heterogeneous somatic mutational landscape of HPV-negative tumors, necessitating divergent analytical platforms and yielding distinct clinical utility profiles for MRD detection and recurrence surveillance. We further outline a pragmatic translational pathway focused on assay standardization, particularly for exosomes and CTCs where this foundational work is most urgently needed, integration of complementary multimodal liquid biopsy approaches, and rigorously designed prospective interventional clinical trials to establish clinical utility. Collectively, these efforts aim to transition HNSCC management from reactive, anatomy-based surveillance to proactive, molecularly guided precision oncology, with the potential to improve therapeutic decision-making and patient outcomes.

## 1. Introduction

Head and neck squamous cell carcinoma (HNSCC) originates from the mucosal epithelium of the oral cavity, oropharynx, hypopharynx, and larynx and remains among the top ten most common cancers globally [1,2]. Despite advances in surgery, radiotherapy, and systemic therapy, mortality remains high, particularly among patients who present with advanced-stage disease or develop recurrence after initial treatment. Current surveillance strategies for HNSCC rely predominantly on radiographic imaging modalities, including computed tomography (CT), magnetic resonance imaging (MRI), and positron emission tomography (PET), as well as serial tissue biopsies. However, these approaches are hampered by inherent limitations that are multifactorial in nature: anatomical (e.g., insufficient spatial resolution to detect microscopic change), temporal (e.g., infrequent and non-continuous sampling intervals), and biological (e.g., intratumoral heterogeneity). These constraints highlight a critical unmet clinical need for monitoring tools that are highly sensitive, suitable for repeated longitudinal use, and biologically informative, capable of detecting recurrent disease at its earliest, pre-anatomic stage when the molecular evidence of recurrence is detectable before any structural correlate is visible on conventional imaging and curative intent intervention remains feasible [3].

Liquid biopsy has therefore emerged as a transformative complement to conventional diagnostics. By analyzing tumor-derived materials released into body fluids (especially blood and saliva in HNSCC patients), clinicians gain access to a real-time, minimally invasive portrait of tumor biology and evolution, including genetic and epigenetic alterations, changes in gene expression, emergence of therapy-resistant clones, and shifts in the tumor immune microenvironment. Importantly, liquid biopsies can provide a lead time advantage over standard imaging—detecting molecular relapse weeks to months before radiographic progression becomes apparent—thereby creating a window for earlier salvage therapy or clinical trial enrollment. Three classes of analytes dominate current research: circulating tumor DNA (ctDNA), extracellular vesicles (primarily exosomes) and circulating tumor cells (CTCs). Each captures a different layer of tumor behavior, offering the potential for complementary insights into disease dynamics, resistance mechanisms, and therapeutic response. These analytes hold particular clinical significance in HNSCC, given the tumor propensity to shed biomarkers into accessible biofluids coupled with the invasiveness and morbidity associated with repeated surgical biopsies. Consequently, liquid biopsy-based monitoring for recurrence offers a minimally invasive, dynamic, and potentially clinically impactful strategy to improve patient outcomes. While other promising analytes such as circulating cell-free RNA (cfRNA), tumor-educated platelets, and circulating proteins have shown early potential, this review focuses specifically on ctDNA, exosomes, and CTCs, as these represent the most extensively studied classes in HNSCC to date and those with the most mature evidence base for tissue-of-origin specificity and technical characterization.

**Figure 1:**
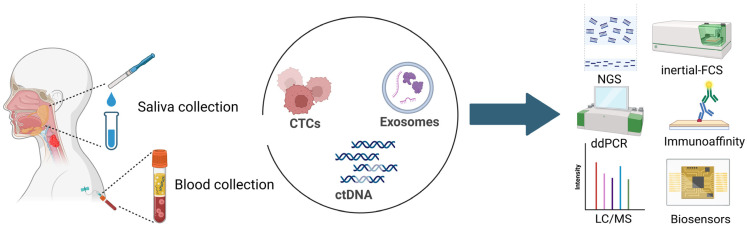
**Integrated liquid biopsy framework for HNSCC.** The schematic illustrates the integration of molecular signals from blood/plasma and saliva for liquid biopsy-based monitoring of HNSCC, spanning the course from pre-diagnosis to post-treatment. The workflow begins with the patient and specimen collection into separate blood/plasma and saliva tubes, followed by analysis of ctDNA, exosomes, and CTCs. The right panel summarizes the primary clinical applications of this multi-analyte, multimodal strategy.

Nonetheless, the translation of liquid biopsy into routine clinical practice has been uneven across different analytes and clinical contexts. This unevenness stems from several factors: differential progress in assay standardization, variable analytical validity across platforms, limited prospective clinical validation, and the fundamental biological differences between HPV-positive and HPV-negative disease that dictate analyte detectability. For instance, HPV-related ctDNA analysis is approaching clinical validation in well-designed observational studies, particularly for minimal residual disease (MRD) detection and recurrence surveillance in HPV-positive HNSCC. However, its predictive utility, the ability to prospectively guide treatment decisions, remains unproven in the absence of completed randomized interventional trials. In contrast, exosome research in HNSCC is best characterized as biologically illuminating but clinically premature; translation to routine clinical practice will require substantial advancements in standardized isolation protocols, reproducible biomarker identification, and prospective validation in well-annotated clinical cohorts. Similarly, CTCs, while offering unique insights into metastatic biology and tumor heterogeneity, remain primarily in the hypothesis-generating phase, hindered by low capture efficiency, lack of standardized detection platforms, and insufficient data linking CTC dynamics to clinical outcomes in interventional settings. This review critically examines the biology, clinical utility, and emerging integrative strategies for ctDNA, exosomes, and CTCs in HNSCC, emphasizing the distinction between prognostic correlation, predictive potential, and true clinical actionability, while acknowledging how viral factors shape their diagnostic and prognostic profiles (Figure 1).

## 2. Biology and Shedding Dynamics

**Figure 2:**
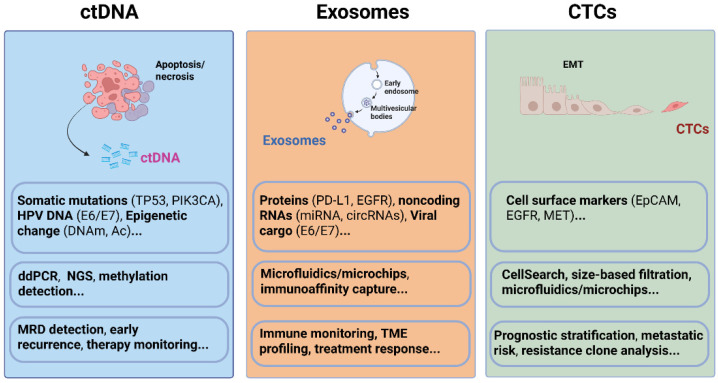
**Leveraging the liquid biopsy analyte triad in HNSCC for precision oncology.** ctDNA, exosomes, and CTCs are characterized by distinct molecular compositions, detectable through both established and emerging methodologies, and have demonstrated clinical utility. These three principal circulating analytes enable multi-parametric, non-invasive tumor profiling to inform precision oncology strategies. DNAm: DNA methylation; Ac: acetylation.

The presence of ctDNA, exosomes, and CTCs in circulation reflects fundamental tumor processes such as cell death, active vesicle secretion, and cellular detachment (Figure 2). Their concentration, molecular composition, and optimal biofluid for detection are influenced by tumor site, volume, vascularity, and HPV status. Understanding how and where these analytes are shed is essential for interpreting assay results and selecting optimal sampling sites.

Anatomic location critically influences the primary route of analyte release into biofluids. Tumors of the oral cavity, directly immersed in the salivary flow, exhibit pronounced shedding of biomarkers, particularly circulating tumor HPV DNA (ctHPV-DNA) and exosomes. For these specific sites, empirical evidence indicates that salivary ctDNA concentrations frequently exceed those in matched plasma samples [4]. Conversely, oropharyngeal tumors often demonstrate a different shedding pattern. Because these sites drain extensively into the lymphatic and systemic circulation, plasma ctDNA typically offers higher diagnostic sensitivity than salivary samples for these regions [4]. Similarly, hypopharyngeal and laryngeal tumors, which lack direct exposure to the salivary flow, predominantly release analytes into the systemic circulation via local capillary beds and lymphatic drainage pathways [5,6]. This spatial heterogeneity provides the biological rationale for dual-fluid profiling, the concurrent analysis of plasma, to capture systemic whole-body tumor burden, and saliva, to enrich for locally derived, tumor-proximal signals, thereby enhancing detection sensitivity, particularly in oral cavity and oropharyngeal carcinomas [7]. However, the practical implementation of salivary liquid biopsy faces several challenges that warrant consideration. Saliva collection, while non-invasive, is subject to significant variability in sample quality due to factors such as time of collection, diurnal variation in salivary flow rate, oral hygiene, food intake, and the presence of inflammatory oral conditions. Furthermore, standardized protocols for saliva processing, storage, and nucleic acid stabilization are not yet universally established, leading to inter-study heterogeneity and difficulties in cross-platform comparison. These pre-analytical variables must be rigorously controlled before salivary assays can be reliably integrated into clinical practice. HPV status further modulates analyte release dynamics. HPV-positive tumors, characterized by reduced stromal desmoplasia and heightened proliferative activity, exhibit more efficient shedding of tumor-derived biomolecules. Moreover, clonal integration of high-copy number viral DNA generates a robust, tumor-specific molecular signature that is absent in HPV-negative malignancies. Importantly, while the biological rationale for dual-fluid profiling is compelling, this approach remains largely investigational. Current evidence is derived predominantly from small, single-institution cohorts with limited prospective validation. The clinical utility of combined plasma and salivary analysis, specifically whether it improves detection sensitivity, provides meaningful lead time over single-fluid approaches, or impacts patient outcomes, has yet to be demonstrated in adequately powered, multicenter prospective trials. As such, dual-fluid profiling should be regarded as a promising but unproven strategy requiring further rigorous investigation before consideration for routine clinical application.

### 2.1. ctDNA

ctDNA enters the bloodstream or saliva predominantly through cellular degradation pathways. Apoptosis generates highly uniform, short DNA fragments, approximately 167 base pairs in length, corresponding to nucleosome-protected DNA. In contrast, necrosis and active secretion release longer, more heterogeneous fragments [8,9]. This fragmentomic profile, including cfDNA fragment size distributions and end-motif patterns, can function as an intrinsic tumor biomarker. ctDNA consistently exhibits a shorter modal fragment length than non-neoplastic cfDNA, a biophysical distinction that enables bioinformatic enrichment without prior knowledge of tumor-specific mutations [9]. These cfDNA fragments collectively reflect the tumor’s clonal architecture at the molecular level, encoding somatic alterations such as point mutations (e.g., TP53, PIK3CA, NOTCH1), copy number variations, and locus-specific methylation signatures [10]. In virally associated HNSCC, particularly HPV-driven oropharyngeal squamous cell carcinoma, ctDNA may also contain clonally integrated, non-human viral DNA sequences. With a short plasma half-life (ranging from ~15 min to several hours), ctDNA provides a near real-time readout of dynamic tumor burden. Accordingly, longitudinal ctDNA monitoring yields kinetic trajectories that closely mirror therapeutic response: effective interventions elicit rapid clearance kinetics, whereas persistent, rising, or rebounding ctDNA levels signal residual disease or early recurrence, often detectable months before radiographic or clinical progression [11,12].

### 2.2. Exosomes

Exosomes undergo active cargo packaging, enabling selective enrichment of molecular constituents, including proteins (e.g., PD-L1, EGFR), lipids, miRNAs, mRNAs, and long noncoding RNAs, that reflect the phenotype of their cell of origin and mediate intercellular communication, immune modulation, and metastatic dissemination [13]. In HNSCC, tumor-derived exosomes function not merely as passive biomarkers but as active effectors of tumor progression and immune modulation. These nanoscale vesicles dynamically remodel the tumor microenvironment through multiple mechanisms, including the promotion of angiogenesis, activation of cancer-associated fibroblasts, and establishment of pre-metastatic niches [14,15]. HPV-positive tumors secrete exosomes containing viral cargo, E6/E7 DNA or mRNA and oncoproteins, which both act as diagnostic markers and participate in immune evasion by degrading tumor-suppressor proteins [16,17]. These virus-laden exosomes thus have dual roles as biomarkers and as active mediators of oncogenesis.

### 2.3. CTCs

CTCs are intact, viable tumor cells that detach from primary or metastatic lesions and enter the bloodstream or lymphatics. Because they retain nuclei, organelles, and complete genomes, CTCs offer greater insight into the heterogeneity and metastatic potential than cell-free biomarkers. Molecular and functional analysis of single CTCs offers unparalleled insights into metastatic heterogeneity, drug resistance mechanisms, and the identification of actionable targets. CTC shedding is an active process often associated with an epithelial-to-mesenchymal transition (EMT), wherein cells lose adhesion molecules like E-cadherin and acquire migratory and invasive properties. HPV status also affects CTC biology, resulting in distinct molecular profiles and differential clinical behavior between HPV-positive and HPV-negative tumors. In HPV-positive HNSCC, many CTCs exhibit partial EMT and lower expression of epithelial markers such as EpCAM, which complicates their capture by conventional antibody-based methods. Label-free or negative-enrichment technologies that rely on size or deformability have proven more effective for these phenotypes [18,19].

## 3. ctDNA for HNSCC Diagnosis and Surveillance

Among liquid biopsy analytes, ctDNA has achieved the most advanced clinical validation in HNSCC, particularly for MRD detection. As these fragments originate directly from tumor cells, they carry somatic mutations, structural variants, methylation signatures, and viral DNA sequences that can serve as highly specific molecular markers in HPV-positive HNSCC.

### 3.1. Clinical Applications

Longitudinal ctDNA surveillance has repeatedly shown prognostic and predictive value. Persistent or rising ctDNA after definitive therapy is associated with shorter progression-free and overall survival, whereas ctDNA clearance correlates with favorable outcomes [8,20,21]. Declines in ctDNA during chemoradiation often precede radiographic response, providing an early indicator of therapeutic efficacy [10,22]. In multiple prospective cohorts (Table 1), tumor-informed assays have detected recurrence months before imaging.

Recent studies have extended the utility of ctDNA monitoring to immunotherapy recipients. The SHIZUKU-HN study prospectively collected serial plasma samples from 27 HNSCC patients undergoing immune checkpoint inhibitor therapy, utilizing the Guardant360 platform to assess variant allele frequency (VAF) and genetic mutations. Mean VAF significantly correlated with tumor volume quantified by 3D CT reconstruction (Spearman’s ρ = 0.70, *p* = 0.001), with ctDNA changes often preceding radiographic progression. Critically, this study identified EGFR and PIK3CA amplifications via ctDNA that were missed in concurrent tissue biopsies, highlighting the ability of liquid biopsy to capture emerging resistance mechanisms and intratumoral heterogeneity not represented in single-site tissue sampling. Analysis of Japan’s C-CAT database (n = 2255) within the same study revealed that ctDNA testing remains underutilized, performed in only 7% of head and neck cancer cases, underscoring the implementation gap despite demonstrated clinical utility [24]. Using tumor-informed personalized ctDNA assay (RaDaR) to monitor 16 patients with recurrent and metastatic HNSCC receiving immune checkpoint blockade [3], ctDNA negativity during treatment was associated with dramatically improved outcomes: disease control (OR 21.7, 95% CI 1.86–754.88), three-year overall survival (HR 0.04, 95% CI 0.00–0.47), and three-year progression-free survival (HR 0.03, 95% CI 0.00–0.37). Conversely, early ctDNA increases correlated with disease progression. The striking hazard ratios, while derived from a small cohort, suggest that ctDNA clearance may serve as a powerful surrogate endpoint for immunotherapy efficacy [10].

Peripheral blood samples were collected preoperatively and one week to five years postoperatively for ctDNA analysis from 16 patients with locally advanced HNSCC. In preoperative samples, ctDNA positivity was 81.3% (13/16), with high-frequency alterations in TP53 (81.3%), CDKN2A copy number reduction (43.8%), and PIK3CA (18.8%). One hypopharyngeal carcinoma patient tested ctDNA-positive 1.5 months postoperatively—6.5 months earlier than radiological recurrence, demonstrating the substantial lead time achievable with sensitive ctDNA monitoring [25]. A paradigm-shifting approach to postoperative MRD detection has recently emerged through analysis of lymphatic fluid rather than plasma. Lazare et al. [26] investigated whether ctDNA in lymphatic exudate collected via surgical drains lymph 24 h after surgery could identify MRD in HPV-independent HNSCC, comparing its performance directly to time-matched plasma. Using an ultra-sensitive tumor-informed sequencing approach across two independent multi-site cohorts (initial n = 36, replication n = 37), the study yielded striking results: lymph ctDNA positivity at 24 h post-surgery was strongly associated with subsequent disease recurrence (initial cohort sensitivity 76%, specificity 63%, log-rank *p* = 0.01; replication cohort sensitivity 65%, specificity 70%, log-rank *p* = 0.04), with enhanced performance for locoregional relapse (sensitivity 78%, specificity 67%, *p* = 0.0004) [26].

Critically, matched plasma collected at this same early timepoint was entirely non-predictive of recurrence (sensitivity 35%, specificity 72%, log-rank *p* = 0.7), demonstrating that lymph represents a proximal biofluid that captures residual disease signals before they reach systemic circulation. This finding has immediate translational implications: postoperative lymphatic drains are routinely placed during neck dissections, yet their fluid is typically discarded. The study suggests this discarded biofluid may contain the earliest, most concentrated molecular evidence of residual disease.

The shedding of ctDNA into circulation is not uniform across tumors and is profoundly influenced by both tumor volume and anatomical location. In a study of patients with locally advanced HNSCC receiving radiochemotherapy, ctDNA levels were correlated with gross tumor volume before treatment start [11]. This relationship underscores that plasma ctDNA sensitivity is volume-dependent, with small primary tumors often falling below the detection threshold.

Tumor necrosis further modulates ctDNA release. Pre-clinical studies in an orthotopic rabbit model of HNSCC have shown that rapid tumor growth followed by auto-necrosis and tumor volume contraction produces a spike in ctDNA levels, suggesting that viable tumor cells may be required for sustained ctDNA release [27]. Highly necrotic tumors shed increased quantities of fragmented DNA into circulation, while well-oxygenated, low-volume tumors may shed minimally, leading to false-negative MRD assessments, a phenomenon termed “biological MRD”, where viable tumor persists but ctDNA remains undetectable [28,29].

In HPV-positive HNSCC, ctDNA analysis is largely simplified to the detection of ctHPV-DNA. Quantitative droplet digital PCR (ddPCR) assays for HPV16 E6/E7 DNA are highly sensitive and specific. Studies show that ctHPV-DNA clearance during radical chemoradiotherapy is an early indicator of treatment response, often preceding radiographic changes by weeks [30,31]. Conversely, persistent or rising post-treatment ctHPV-DNA is a near-definitive marker of residual disease, predicting clinical recurrence with a lead time of 4–6 months over imaging [32,33]. This enables risk stratification: patients with undetectable post-treatment ctHPV-DNA have an exceptionally low risk of relapse and are ideal candidates for de-escalation protocols.

Due to lack of a universal viral marker in HPV-negative HNSCC, tumor-informed assays are the gold standard [34]. This approach entails whole-exome or targeted panel sequencing of the primary tumor to identify patient-specific somatic mutations, such as those in TP53, NOTCH1, PIK3CA, and FAT1. Subsequently, personalized detection assays, either ddPCR or mutation-specific NGS, are developed to monitor these variants in serial plasma samples. Postoperative or post-therapeutic detection of such tumor-derived mutations is robustly associated with significantly shorter recurrence-free survival and overall survival. In contrast, tumor-agnostic approaches leverage comprehensive NGS panels or DNA methylation profiling to detect cancer-associated molecular signals without requiring prior tumor tissue sequencing; these methods enable a more streamlined workflow and have demonstrated promising utility for prognostic risk stratification in ongoing prospective clinical studies.

Beyond HNSCC, compelling evidence from hematologic malignancies reinforces the broad applicability of ctDNA-based MRD monitoring. A prospective longitudinal study by Liu et al. [35] in 50 children with acute myeloid leukemia (AML) demonstrated that peripheral blood ctDNA monitoring achieves 92.8% concordance with bone marrow tumor DNA sequencing, with ctDNA dynamics serving as a powerful prognostic indicator. Patients who cleared ctDNA after three cycles of chemotherapy exhibited progression-free survival similar to those with persistently negative ctDNA (*p* = 0.728), suggesting that treatment de-escalation may be feasible in molecular responders. Notably, patients achieving > 3 log reduction in ctDNA without complete clearance maintained favorable outcomes comparable to those with complete clearance (*p* = 0.564), challenging the binary classification of MRD positivity and introducing the concept of “molecular response depth” as a continuous variable for risk stratification [35].

Despite the compelling nature of these findings, a critical appraisal of the literature reveals significant caveats that temper generalizability. As summarized in Table 1, many of the pivotal studies, particularly in HPV-negative cohorts, are limited by small sample sizes (often <50 patients) and a predominantly retrospective design [21,23]. Although prospective studies tend to enroll larger cohorts, they remain largely observational and lack an interventional component wherein ctDNA results actively inform clinical decision-making or treatment allocation. To date, no randomized trial has demonstrated that ctDNA-guided surveillance or treatment escalation/de-escalation improves overall survival in HNSCC, the highest level of evidence required for practice-changing recommendations. Moreover, the reported performance metrics must be interpreted with caution. For example, while the TTMV-HPV DNA assay boasts impressive positive and negative predictive values (95%) in a real-world setting [24], these values are highly dependent on the underlying disease prevalence in the cohort and may not be replicable in lower-risk populations. The confidence intervals around these point estimates are often wide, reflecting statistical uncertainty. Crucially, even the most sensitive assays face the biological limitation of “biological MRD”, where tumor cells are dormant or located in a sanctuary site, which does not shed ctDNA into the circulation, leading to false-negative results. Therefore, while ctDNA is arguably the most clinically mature liquid biopsy analyte in HNSCC, its role as a practice-changing, rather than simply prognostic, tool awaits validation in large-scale, multicenter interventional trials (e.g., trials randomizing patients to ctDNA-guided surveillance vs. standard imaging).

### 3.2. Analytical Approaches

A key limitation of ctDNA analysis is its scarcity in circulation and the overwhelming background of non-tumor cfDNA. The concentration of tumor ctDNA can be as low as 0.01% of total cfDNA, especially in early-stage or minimal residual disease settings [36]. As a result, sensitive enrichment approaches are critical to filter out cfDNA and preserve ctDNA. There are some existing methods to enrich and isolate ctDNA. One method to enrich ctDNA is pre-analytical control. Blood must be collected in tubes that inhibit leukocyte lysis and processed promptly to avoid genomic DNA contamination. Stabilizing tubes such as Streck or PAXgene and double-centrifugation protocols can be used to minimize contamination with genomic DNA [37]. Extraction typically employs silica-column or magnetic bead-based kits, which enable capture of nucleic acids from plasma through selective binding. However, variability in extraction yield and fragment-size bias across platforms mandates laboratory-specific validation. Comparative studies have shown that certain commercial kits, such as the QIAamp Circulating Nucleic Acid Kit, yield superior recovery and less high-molecular-weight contamination [38]. Furthermore, fragment-length selection can enrich tumor DNA because ctDNA tends to be shorter (90–150 bp) than background cfDNA. Computational or physical size-selection (in favor of shorter fragments) increases mutant allele detection twofold or more [39,40]. However, aggressive filtering can reduce overall yield, requiring optimization of fragment-size thresholds to balance enrichment with recovery.

Technological innovations are rapidly democratizing ctDNA analysis through portable, automated platforms. Yin et al. [41] recently developed a handheld, fully automated, rotating magnetic field-driven integrated tube system with sample-in–answer-out capability for nucleic acid testing. This compact device (0.76 kg, 10 × 10 × 10 cm) integrates sample lysis, purification, elution, PCR amplification, and detection within a single disposable tube, completing the entire workflow in under one hour. Validation for hepatitis B virus detection achieved sensitivity of 5 copies/μL with a linear range of 5 × 10^1^ to 5 × 10^5^ copies per reaction. This platform addresses several barriers to ctDNA implementation in HNSCC: the need for centralized laboratory infrastructure, complex multi-step workflows requiring trained personnel, and high per-sample costs. For resource-limited settings where HNSCC burden is often highest, such point-of-care technologies could enable serial ctDNA monitoring without requiring sophisticated molecular diagnostics facilities. The rotating magnetic field design eliminates the need for external pumps or valves, reducing mechanical complexity while maintaining extraction efficiency, a critical consideration for adapting tumor-informed HNSCC assays to community hospital settings [41].

Beyond physical separation, molecular enrichment methods target tumor-specific alleles during library preparation and amplification. Hybridization capture technologies, such as Cancer Personalized Profiling by Deep Sequencing (CAPP-Seq), use biotinylated probes complementary to known cancer-associated regions, allowing selective selection of relevant fragments [42,43]. This approach achieves broad genomic coverage while maintaining high analytical sensitivity. Combining CAPP-Seq with a digital error suppression framework further improved its analytical sensitivity, enabling reliable detection of ctDNA down to 0.0025% mutant allele frequency using as few as 30 tumor-specific mutations [43]. Alternatively, multiplexed amplicon-based strategies, including tagged amplicon sequencing, target predefined genomic loci and incorporate unique molecular identifiers to mitigate PCR- and sequencing-related errors [44]. Pre-PCR mutation-enrichment methods, such as NaME-PrO and DEASH, selectively eliminate wild-type templates or exploit mismatch-sensitive enzymatic activity to preferentially amplify rare mutant molecules [45].

### 3.3. Challenges and Perspectives

Despite its considerable clinical promise, ctDNA-based MRD assessment faces several challenges that impede its widespread implementation. Tumor-informed assays necessitate adequate quantities of high-quality archival tumor tissue, a resource that is frequently unavailable or insufficient. Analytical sensitivity varies across platforms and laboratories, and clinically validated, standardized thresholds for MRD positivity remain to be prospectively established. Moreover, the cost and technical complexity of NGS constrain accessibility in resource-limited healthcare settings. Biologically, factors such as low tumor DNA shedding rates or the presence of biologically dormant tumor cells, termed biological MRD, may result in false-negative ctDNA findings. The convergence of hematology–oncology liquid biopsy research offers valuable lessons for HNSCC. A 2025 review by Butler et al. highlighted that peripheral blood-based MRD detection in AML is now recommended by the European Leukemia Net for certain genetic abnormalities, enabling more frequent testing intervals and better temporal resolution of malignant expansion while sparing patients invasive bone marrow procedures [46]. A recent systematic review provides crucial context for interpreting the evidence base. Jones et al. [47] systematically evaluated 18 studies investigating ctDNA for treatment response prediction in HNSCCs. While ctDNA monitoring demonstrated high negative predictive value (91.7%) for residual or recurrent disease, the review identified significant limitations in the existing literature. The median REMARK score was 12.65 out of 20, indicating suboptimal study quality. Median cohort size was only 59 patients, and median follow-up was 24.5 months. Most studies (13/18) investigated HPV ctDNA alone, leaving HPV-negative disease comparatively understudied. Importantly, consecutive testing mitigated positive predictive value variability, suggesting that serial rather than single timepoint sampling may be essential for reliable clinical implementation [47]. This systematic appraisal underscores that while the prognostic signal of ctDNA is robust, the field requires larger, higher-quality prospective studies with standardized methodologies. Beyond study design considerations, fundamental biological factors may limit ctDNA sensitivity. Low tumor DNA shedding rates, particularly in certain histologic subtypes or after effective therapy, can yield false-negative results. Additionally, the phenomenon of biological MRD represents an inherent limitation of ctDNA-based surveillance [47].

A critical and underappreciated biological confounder in ctDNA-based liquid biopsy is clonal hematopoiesis of indeterminate potential (CHIP), which refers to the age-related expansion of hematopoietic stem cells carrying somatic mutations in leukemia-associated genes (e.g., DNMT3A, TET2, ASXL1) without meeting criteria for hematologic malignancy [48]. These mutations, present at variant allele frequencies (VAF) ≥ 2%, are released into the circulation from white blood cells and can be mistakenly identified as tumor-derived ctDNA [49,50]. CHIP-associated mutations—particularly in TP53, a gene frequently mutated in HPV-negative HNSCC, can generate false-positive signals that mimic molecular residual disease or suggest the presence of targetable alterations. Studies in late-stage non-small cell lung cancer have shown that paired peripheral blood mononuclear cell (PBMC) sequencing analysis may be needed to remove CHIP variants for comprehensive genomic profiling using plasma samples to identify true somatic mutations [51]. The number of CHIP variants is positively associated with age, and TP53 is the most frequently mutated gene in CHIP [52]. This poses a particular challenge for HPV-negative HNSCC, where TP53 mutations are both common in tumors and frequently encountered as CHIP variants.

Ultimately, large-scale, prospective, multicenter interventional trials, particularly those in which therapeutic decisions are prospectively guided by ctDNA MRD status, are essential to transition ctDNA from a prognostic biomarker to a clinically actionable predictive tool.

## 4. Exosomes for HNSCC Diagnosis and Surveillance

Exosome analysis extends beyond genomic profiling to encompass proteomic and transcriptomic characterization of tumor-derived cargo, thereby providing insights into tumor–microenvironment crosstalk and enabling the study of functional intercellular communication. Several studies demonstrate that plasma or salivary exosomal profiles correlate with disease burden and therapeutic response (Table 2). Theodoraki et al. reported that PD-L1-positive exosomes declined within weeks of therapy, suggesting their potential as early response markers [53]. Ludwig et al. likewise observed that exosome concentration and immunosuppressive activity were higher during active disease than no evident disease, with elevated PD-L1 cargo inhibiting CD8^+^ and NK-cell function in co-culture experiments [54]. These findings indicate that exosomes mirror both tumor load and immune modulation, and that their cargo can be leveraged to study how tumors systemically influence immune cell activity.

### 4.1. Clinical Applications

Exosomal cargo represents a rich and biologically informative source of biomarkers. A number of studies showed that exosomal miRNAs have important diagnostic utility. Wang et al. showed that exosomal miR-21 discriminated laryngeal squamous cell carcinoma from healthy controls with high sensitivity and specificity [61], while He et al. identified miR-24-3p as a salivary biomarker for oral squamous carcinoma (AUC = 0.738) [56]. Protein cargo such as EGFR and HSP70 in plasma-derived exosomes correlate with advanced stage and poor outcome [62,63], and elevated exosomal miR-196a predicts recurrence risk and reduced overall survival [64].

Beyond their diagnostic potential, exosomes show promise as biomarkers of treatment response: circulating exosomal PD-L1 levels track chemotherapy response and disease activity [65], while other exosomal markers, such as CD16, correlate with tumor stage and invasion, further linking EV phenotype to disease aggressiveness [66]. Additionally, exosomal PD-L1 serves as a dynamic, quantifiable indicator of immune checkpoint activity; its levels correlate with tumor burden and demonstrate predictive value for both response to and resistance against anti-PD-1/PD-L1 immunotherapies [67]. Radiotherapy studies similarly suggest that changes in exosomal miRNA profiles, such as increases in miR-9 may indicate enhanced radiosensitivity in HPV-positive HNSCC [68]. In a 40-patient OSCC cohort, exosomal mRNAs were quantified in paired saliva and serum, and a two-gene salivary panel (TNF-α + OAZ1) achieved an AUC of 0.89 (80% sensitivity, 90% specificity) for distinguishing OSCC from healthy controls; several other salivary exosomal mRNAs (MMP9, IL8, S100P, SAT, OAZ1) differentiated grade I compared with grade III disease, and IL6 levels correlated with lymph-node metastasis [69]. These studies demonstrate that exosomal mRNA panels can provide noninvasive insights into both diagnosis and tumor aggressiveness, and with further validation, such biomarkers could enable real-time treatment adaptation. Recent studies have substantially expanded our understanding of exosomal cargo as functionally relevant biomarkers. Hofmann et al. [58] comprehensively characterized saliva-derived exosomes from HNSCC patients (n = 21) compared to healthy donors (n = 12), revealing that salivary exosomes carry high levels of CD44v3, PD-L1, and CD39. Critically, saliva was significantly richer in tumor-derived (CD44v3+) exosomes and poorer in hematopoietic cell-derived (CD45+) exosomes compared to plasma, establishing saliva as a proximal biofluid enriched for tumor-specific signals. Functional assays demonstrated that saliva-derived exosomes from HNSCC patients attenuated CD8+ T cell activity and produced high levels of immunosuppressive adenosine. miRNA profiling identified 62 healthy donor-exclusive and 31 HNSCC-exclusive miRNAs, with pathway analysis implicating RAS/MAPK, NF-κB, Smad2/3, and IFN-α signaling [58].

Comparative analysis of biofluids reveals complementary information. Hofmann et al. [59] directly compared plasma- and saliva-derived exosomal miRNA profiles from 11 HNSCC patients and 5 healthy donors, identifying 119 miRNAs that overlapped between biofluids. From these, 29 tumor-exclusive miRNAs associated with TP53, TGFB1, PRDM1, FOXO1, and CDH1 signaling were selected. The top 10 miRNA candidates with the strongest correlation emerged as diagnostic panels to discriminate cancer from healthy individuals and as potentially prognostic panels for disease-free survival. Importantly, exosomal miRNAs were differentially represented in HPV-positive versus HPV-negative disease and across tumor stages, suggesting that dual-fluid profiling may enhance diagnostic accuracy [59]. Immune modulation via exosomal surface molecules represents an emerging theme. Theodoraki et al. [60] demonstrated that plasma-derived CD16+ exosomes significantly correlate with the abundances of CD16+ non-classical monocytes and CD16+ intermediate monocytes in HNSCC patients. Significant correlations were also observed between CD16+ exosomes and adhesion molecules CD29 (integrin β1) and CX3CR1 on monocyte subsets, suggesting that exosomal phenotypes may serve as surrogates for the systemic immune landscape. This positions exosomes not merely as tumor-derived cargo carriers but as integrative biomarkers reflecting tumor-immune crosstalk [60].

Although the diagnostic and longitudinal monitoring potential of exosomes in cancer is increasingly recognized, the field remains hampered by pronounced methodological heterogeneity and insufficient analytical validation, which critically impede cross-study comparability and hinder clinical translation. As outlined in Table 2, the majority of current studies are early-phase, hypothesis-generating investigations, frequently underpowered due to small sample sizes (n < 50). For example, a study reporting that salivary exosomal miR-24-3p discriminates patients with oral squamous cell carcinoma from healthy controls with an AUC of 0.738 [44] yields preliminary promise; however, this performance falls below the commonly accepted threshold for clinical diagnostic utility (AUC > 0.80), and the absence of reported confidence intervals precludes robust assessment of statistical reliability. More fundamentally, critical pre-analytical variables—including biospecimen collection timing, storage conditions, centrifugation parameters, and, most notably, exosome isolation methodology (e.g., ultracentrifugation, size-exclusion chromatography, or microfluidic platforms), exert profound effects on vesicle yield, purity, integrity, and molecular cargo composition [60]. As a result, assay outcomes—including sensitivity, specificity, and biomarker abundance—are highly methodology-dependent, limiting inter-laboratory reproducibility and generalizability. Until the field implements rigorously harmonized protocols aligned with the Minimal Information for Studies of Extracellular Vesicles (MISEV2018) guidelines [55,70], and advances beyond single-center discovery efforts toward large-scale, pre-registered, multicenter analytical and clinical validation studies, exosome-based biomarkers will remain investigational tools rather than validated, clinically actionable assays. Exosome research in HNSCC is best characterized as biologically illuminating but clinically premature. While the mechanistic insights into tumor-immune crosstalk and the identification of candidate biomarkers are valuable for guiding future research, translation to routine clinical practice will be required.

### 4.2. Analytical Approaches

Exosome isolation remains technically challenging, partly because EVs overlap in size and biophysical characteristics with lipoproteins and protein aggregates. The MISEV2018 guidelines emphasize that isolation methods must be selected based on the intended downstream application and must transparently report EV purity [70]. Conventional ultracentrifugation and density-gradient flotation remain widely used, but both risk co-pelleting contaminants and can generate shear-induced vesicle deformation. Size-exclusion chromatography preserves vesicle integrity but often requires additional concentration steps. Using direct immunoaffinity capture, Jeppesen et al. demonstrated that many proteins and extracellular RNAs once thought to be inside exosomes, such as Argonaute 1–4, are actually present in non-vesicular fractions, indicating that standard ultracentrifugation pellets contain a mixture of bona fide small EVs and free protein-RNA complexes [71].

To overcome throughput limitations, next-generation platforms combine microfluidics and acoustofluidics to separate EVs based on size, compressibility, or surface markers. For example, Wang et al. developed a microfluidic chip that isolates EV-associated HPV-16 DNA directly from saliva and enables rapid nucleic-acid detection, achieving 90% sensitivity and 94% specificity for HPV-16 E6/E7 DNA in oropharyngeal cancer. Similar innovations have been reported. Wu et al. employed surface acoustic waves for rapid, label-free separation of nanoscale vesicles [72], and Zhao et al. demonstrated an integrated “ExoSearch” immunomagnetic chip that simultaneously quantifies tumor-associated exosomal proteins in plasma from ovarian cancer patients [73]. Biophysical characterization contributes another discriminative layer. Tumor-derived exosomes often display altered membrane stiffness, zeta potential, and size distributions. Hoshino et al. found that cancer-derived vesicles possess distinct protein, lipid, and RNA signatures that classify tumor type with high accuracy [74]. Together, these mechanistic and methodological advances underscore exosomes as a versatile component of the liquid biopsy toolkit.

### 4.3. Challenges and Perspectives

Methodological variation remains a barrier to clinical translation. Different protocols enrich different extracellular vesicle subsets and contaminants. In saliva-based HNSCC studies, direct experimental comparisons have shown differences. One optimization study found that multiple ultracentrifugation protocols and size-exclusion chromatography all yielded morphologically valid small EVs, but with substantial differences in particle size distribution, particle-to-protein ratio, and recovery, and concluded that there is “no common consensus on the ideal method” for salivary EV isolation [75]. Ultracentrifugation and size exclusion chromatography gave the highest particle-to-protein yields; however, size exclusion chromatography recovered markedly fewer vesicles, illustrating how technical choices can skew the apparent EV concentration and cargo and complicate cross-study cut-offs for “positive” exosomal tests [75].

Biological heterogeneity is a second key limitation. Saliva and plasma both contain mixtures of tumor-derived EVs and vesicles from non-tumor cells. In salivary samples, abundant proteins such as α-amylase and mucins can mask low-abundance EV proteins and interfere with downstream assays [75]. Even in carefully processed samples, the relative proportions of tumor-derived vs. non-tumor EVs vary with tumor site, stage, and local inflammation. This makes it unlikely that bulk measurements of “total exosome concentration” will provide sufficient specificity; future assays will probably need to integrate cell-of-origin markers (e.g., CD3 vs. tumor-enriched CD44v3 subsets) [58], cargo signatures (miRNA/mRNA/proteins), and possibly biophysical properties into multiplex panels rather than relying on single universal exosomal markers.

Exosomal cargo is not exclusively tumor-derived; inflammatory and infectious conditions common in HNSCC patients, including periodontitis, mucositis, tobacco-associated chronic inflammation, and postoperative wound healing, can profoundly alter exosomal composition. Salivary exosomes, in particular, are contaminated by abundant proteins (α-amylase, mucins) and microbial vesicles that mask low-abundance tumor-specific signals [76].

Recent work has demonstrated that saliva-derived exosomes from HNSCC patients carry high amounts of CD44v3, PD-L1, and CD39, and are significantly richer in tumor-derived (CD44v3+) exosomes compared to plasma [58]. In the context of active inflammation, exosomal cargo reflecting these pathways may be elevated independent of tumor burden, confounding interpretation of tumor-derived signals. The composition of tumor-derived exosomes is dynamically modulated by the surrounding microenvironment. Hypoxic conditions, common in HNSCC due to rapid tumor growth and aberrant vasculature, enhance the release of exosomes. Similarly, chemotherapy and radiation therapy increase exosome shedding. Immunotherapy further complicates exosomal interpretation. Exosome-associated PD-L1, secreted by both tumor and immune cells, can suppress T cell function and may increase paradoxically during pseudo-progression, complicating response assessment.

These constraints suggest that progress will come from making EV assays’ workflow defined, rather than treating exosomes as an independent analyte with universal cutoffs. Because isolation strategies influence analyte recovery and concentration, the establishment of clinically defensible thresholds will require transparent reporting of preanalytical handling, separation methodologies, and downstream characterization to ensure reproducibility and comparability across sites [70].

## 5. CTCs for HNSCC Diagnosis and Surveillance

CTCs provide a direct cellular representation of the tumor and offer the potential for single-cell genomic and phenotypic analyses, although this remains technically challenging with high failure rates due to low CTC purity, limited input material, and the risk of amplification bias or allele dropout [77,78].

### 5.1. Clinical Applications

The simple enumeration of CTCs (≥1 CTC per blood volume) has prognostic value in HNSCC, associating with advanced stage, metastatic disease, and poorer survival. Several investigations have established the prognostic significance of CTC detection in HNSCC. In a cohort of 154 patients, Zhang et al. found that baseline CTC positivity, detected by spiral microfluidics, strongly predicted recurrence and mortality (*p* < 0.0001) [79]. Persistently detectable CTCs after therapy correlated with a 2.5-fold higher likelihood of relapse or death (*p* < 0.05). A meta-analysis confirmed that CTC presence was linked to shorter disease-free survival [80], while Sun et al. observed higher recurrence and progression rates among CTC-positive patients [81]. Recent studies have substantially refined our understanding of CTC heterogeneity and clinical significance. Chikamatsu et al. [82] comprehensively characterized CTCs based on epithelial-related marker expression in 60 CTC-positive HNSCC patients, revealing that 80.0% were EPCAM-positive, 33.3% EGFR-positive, and 51.7% MET-positive. Critically, CTCs expressing distinct markers showed differing EMT and immune-regulatory gene expression profiles. EPCAM+ CTCs were associated with advanced-stage disease, while EGFR+ CTCs correlated with locoregional relapse and significantly shorter progression-free survival (*p* = 0.007; hazard ratio = 3.254). Patients with EPCAM/EGFR double-positive CTCs exhibited the poorest prognosis, underscoring the importance of multi-marker approaches for capturing clinically relevant CTC subpopulations [82]. Additionally, because CTCs can be measured serially, they provide a dynamic window into treatment response. In HNSCC, emerging evidence suggests that CTC dynamics correlate with clinical outcomes. For example, Poellmann et al. evaluated changes in CTC counts over time in patients undergoing therapy and reported that, in HPV-negative cases, CTC counts declined significantly during treatment, whereas patients who experienced recurrence exhibited persistently elevated CTC levels during and after therapy [83].

Although CTCs consistently demonstrate prognostic significance across multiple studies, their clinical translation remains constrained by two interrelated challenges: substantial technological heterogeneity in isolation and detection methodologies, and the inherently low biological abundance of CTCs in peripheral blood. While meta-analyses confirm a statistically robust association between CTC enumeration and clinical outcomes [62], this prognostic signal is derived from highly heterogeneous studies, varying markedly in patient selection criteria, timing of blood collection (e.g., pre-treatment vs. on-treatment), and analytical platforms used for CTC enrichment and identification. For instance, the claim that baseline CTC positivity “strongly predicted recurrence” [61] must be interpreted cautiously in light of empirical evidence indicating that even state-of-the-art microfluidic enrichment technologies detect CTCs in only 54–57% of patients with recurrent or metastatic disease [69,70]. This suboptimal sensitivity poses a fundamental limitation: a negative CTC result cannot be interpreted as definitive evidence of absence of residual or disseminated tumor cells, thereby undermining its reliability for guiding therapeutic decisions.

Moreover, the choice of detection methodology introduces systematic biological bias in the CTC subpopulations captured. Preclinical xenograft studies have shown that EpCAM-dependent platforms, such as the FDA-cleared CellSearch^®^ system, systematically underestimate total CTC burden by failing to isolate EMT-associated CTCs, which frequently downregulate EpCAM expression [71]. In contrast, label-free approaches (e.g., size-based or density-based isolation) capture a broader phenotypic spectrum of CTCs but suffer from a lack of analytical standardization across laboratories, precluding the establishment of universally applicable, clinically validated thresholds for CTC positivity. Consequently, emerging observations, such as the association between PD-L1 overexpression on CTCs and inferior survival outcomes [72], remain hypothesis generating. Although these findings suggest a plausible mechanistic link and potential utility of PD-L1+ CTCs as predictive biomarkers for immune checkpoint inhibitor response, they currently reflect correlative associations rather than experimentally confirmed causal relationships.

CTC analysis in HNSCC currently offers validated prognostic information in research settings but lacks the analytical standardization, regulatory approval, and interventional trial evidence required for routine clinical use. The biological insights, particularly regarding phenotypic heterogeneity and EMT, are valuable for guiding future assay development and therapeutic targeting.

### 5.2. Analytical Approaches

Several approaches are being pursued to isolate and enrich CTCs to overcome the current limitations. Broadly, these approaches can be classified into label-dependent and label-independent methods, each exploiting different cellular properties such as surface marker expression, size, deformability, or electrical characteristics.

The most established system, CellSearch^®^, relies on immunomagnetic separation and is the only FDA-cleared method for CTC enumeration in several metastatic cancers. It isolates cells expressing the epithelial cell adhesion molecule (EpCAM), followed by cytokeratin staining and CD45-negative selection to exclude leukocytes. However, the system’s reliance on epithelial markers limits sensitivity for CTCs that have undergone EMT and downregulated EpCAM, a phenomenon more common in HPV-positive or advanced-stage tumors [84]. This approach enables both positive and negative selection modes, allowing recovery of viable, antigenically diverse tumor cells suitable for single-cell RNA sequencing and culture. In HNSCC, where CTCs frequently display hybrid epithelial–mesenchymal phenotypes, the label-free CTC-iChip offers distinct advantages by preserving cellular heterogeneity and enabling capture of clinically relevant subpopulations. Another label-free strategy, the Vortex CTC chip, exploits inertial microfluidics to selectively trap larger and less deformable tumor cells within microscale vortices under controlled flow conditions [85]. The captured cells can be released intact and viable, facilitating downstream analyses such as RNA sequencing or ex vivo drug testing. In proof-of-concept studies, Vortex technology achieved CTC enrichment within minutes and demonstrated strong compatibility with live-cell assays. Zhang et al. [86] recently developed an innovative CTC isolation platform combining tetrahedral DNA nanostructure-based trivalent aptamer magnetic nanospheres (TDF-Cocktail-MNP) with a magnetic microfluidic separation device. This system simultaneously presents three aptamers: EpCAM targets epithelial CTCs, vimentin captures mesenchymal types, and EGFR binds to cells overexpressing EGFR. This multi-target approach enables efficient isolation of heterogeneous CTC populations from whole blood samples while preserving cell viability through reduced mechanical stress. Captured CTCs are enriched using the IsoFlux System, and after nuclease-mediated removal of the DNA scaffold, cells can be classified and quantified through immunocytochemistry. Validated with clinical samples, this platform shows strong potential for capturing phenotypically diverse CTCs and enabling reliable downstream analysis [86].

**Figure 3:**
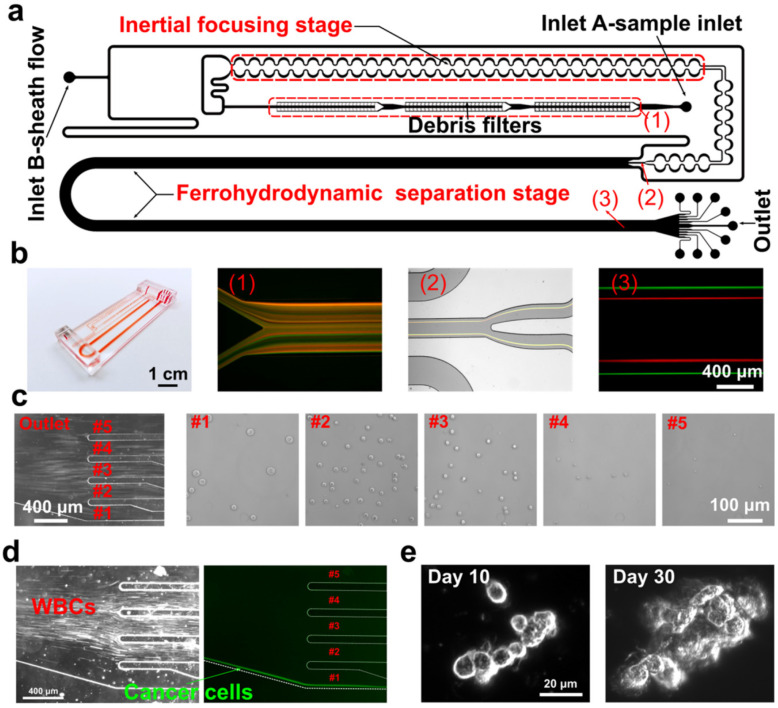
**Overview of inertial-FCS.** (**a**) Schematic drawing of the inertial-FCS. (**b**) A photo and operation of inertial-FCS microchannel. Red: 6 µm beads; Green: 10 µm beads (1) Particles prior to inertial focusing. (2) Particles after inertial focusing but before ferrohydrodynamic separation. (3) Particles after ferrohydrodynamic separation. (**c**) Bright field images of cells separation based on diameters. (**d**) Cancer cell separation using inertial-FCS device. (**e**) Bright field images of cultured CTCs at day 10 and day 30. CTCs are separated from patients with non-small cell lung cancer. *Reproduced from Ref.* [87] *with permission from the Royal Society of Chemistry.*

More recently, inertial–ferrohydrodynamic cell separation (inertial-FCS) has emerged as an advanced microfluidic platform that combines fluid dynamics with optical detection to isolate CTCs (Figure 3) [87]. Unlike traditional methods, it selects a label-free method based on the size difference between CTCs and white blood cells rather than molecular markers. Inertial-FCS can differentiate the cells based on their diameter with a resolution of 1–2 µm. This system allows continuous, high-throughput CTC separation with minimal shear stress and can integrate directly with downstream molecular profiling modules, enabling real-time analysis. However, it is important to note that while other label-free methods such as the CTC-iChip and Vortex CTC chip have been evaluated in multiple HNSCC cohorts and across several laboratories [61,67,70], the inertial-FCS platform has thus far been described primarily in proof-of-concept studies with limited validation in clinical HNSCC samples.

### 5.3. Challenges and Perspectives

One major limitation is the rarity of CTCs and the resulting technical complexity of their detection. Typical reported concentrations range from 1 to 100 CTCs per milliliter of blood in solid tumors, necessitating highly sensitive enrichment strategies and meticulous sample handling [88]. Even in optimized HNSCC cohorts, detection is incomplete: spiral microfluidic enrichment identified CTCs in only 54% of patients in a pilot study [89], and EpCAM-based OncoDiscover technology detected baseline CTCs in 57% of recurrent/metastatic cases [88]. Heterogeneous detection rates across platforms and disease stages challenge the designation of CTC-negative status and may conceal residual disease in some patients. Platform heterogeneity is another barrier to clinical use. Label-dependent systems such as CellSearch^®^ provide standardized reagents and scoring criteria but are intrinsically biased toward EpCAM-high, epithelial CTCs and may miss EMT-shifted or hybrid cells. This has been noted in a xenograft breast cancer model. Gorges et al. showed that an EpCAM-based assay failed to detect CTCs in vivo despite visible metastases because CTCs downregulated EpCAM and upregulated mesenchymal markers, concluding that sole reliance on epithelial markers for CTC enrichment may result in an underestimation of the total CTC burden, particularly for cells undergoing EMT [90]. Conversely, label-free microfluidic devices enrich CTCs based on size and deformability and can capture a broader phenotypic spectrum, but they require more complex downstream staining and are not yet harmonized across manufacturers. As a result, thresholds for CTC positivity, definitions of clusters, and the panels used to assign epithelial, mesenchymal, immune-regulatory, or HPV-related phenotypes differ substantially across studies, limiting cross-trial comparability.

Although CTCs clearly provide prognostic and pharmacodynamic information, they have not yet been integrated into treatment-altering algorithms in HNSCC. Liquid biopsy tests that capture CTCs in whole blood to assess prognosis in patients with metastatic breast, prostate, or colorectal cancer were approved by the FDA [91,92]. A research group in Australia recently utilized a spiral microfluidics-based CTC isolation platform to systematically assess the clinical utility of CTCs in 154 HNSCC patients for cancer prognostication and post-treatment monitoring [79]. Their findings indicate that early detection of CTCs enables more precise risk stratification, which may inform therapeutic decision-making and potentially improve patient outcomes. However, multi-center clinical trials are still required to fully define the role of CTCs in optimizing treatment strategies and predicting therapeutic responses. Likewise, PD-L1-overexpressing CTCs at the end of curative-intent treatment independently predicted shorter progression-free and overall survival, and Strati et al. explicitly suggested that adjuvant PD-1 inhibitors should be evaluated in this high-risk subgroup, but such trials have not yet been completed [93].

## 6. HPV Status as a Central Determinant in HNSCC Liquid Biopsy

HPV status represents the most critical biological variable in HNSCC, fundamentally altering tumor biology, clinical behavior, and liquid biopsy analyte profiles. HPV-positive HNSCC is now recognized as a distinct disease entity, characterized by unique molecular pathogenesis, favorable prognosis, and differential treatment responses compared to HPV-negative HNSCC [94]. This dichotomy has profound implications for liquid biopsy assay design, biofluid selection, and clinical interpretation.

HPV-positive HNSCC exhibits biological features that enhance shedding of tumor-derived materials into circulation. Reduced stromal desmoplasia, increased proliferative activity, and altered apoptotic pathways contribute to more efficient release of ctDNA, exosomes, and CTCs [58]. Critically, the clonal integration of high-copy number viral DNA generates a robust, tumor-specific molecular signature that is completely absent in HPV-negative malignancies [95]. This provides an amplification effect: each tumor cell releases multiple copies of viral DNA, substantially increasing the concentration of ctHPV-DNA in plasma and saliva compared to the single-copy somatic mutations that must be tracked in HPV-negative disease.

HPV-positive disease enables simplified detection via ultra-sensitive digital PCR assays targeting HPV16 E6/E7 DNA, achieving analytical sensitivity down to 0.001% variant allele frequency. Large cohort studies demonstrate pretreatment ctHPV-DNA detection rates of 89–95%, with levels strongly correlated with nodal stage and tumor volume. In contrast, HPV-negative HNSCC requires tumor-informed approaches using next-generation sequencing to identify patient-specific somatic mutations (e.g., TP53, NOTCH1, PIK3CA, FAT1), followed by personalized ddPCR or NGS monitoring. This fundamental difference in assay complexity has direct implications for cost, turnaround time, and accessibility.

HPV-positive tumors secrete exosomes carrying viral cargo, including E6/E7 DNA, mRNA, and oncoproteins, which serve as highly specific diagnostic markers. These virus-laden exosomes participate in immune evasion by degrading tumor-suppressor proteins and modulating the tumor microenvironment. HPV-negative exosomes, by contrast, contain predominantly host-derived miRNAs and proteins related to inflammation and EMT, lacking a universal tumor-specific signature.

HPV status profoundly influences CTC phenotype and detectability. In HPV-positive HNSCC, CTCs frequently exhibit partial EMT and lower expression of epithelial markers such as EpCAM, complicating capture by conventional antibody-based methods. p16-positive CTCs are detectable in approximately 51% of patients and serve as independent prognostic factors, with p16 positivity paradoxically associated with better overall survival despite higher progression risk, reflecting the favorable prognosis of HPV-positive disease [82]. HPV-negative CTCs demonstrate higher EpCAM and EGFR expression, with EGFR+ CTCs correlating with locoregional relapse and significantly shorter progression-free survival (HR 3.254, *p* = 0.007).

Anatomic site preferences for HPV-positive tumors (primarily oropharynx) versus HPV-negative tumors (oral cavity, larynx, hypopharynx) dictate optimal biofluid selection. A comparative analysis of TP53-mutated tumor DNA in 68 oral cavity SCC patients revealed that while mutated ctDNA was identified in 68% of tumors, concordance between saliva and plasma was only 29.4%, indicating that these biofluids capture distinct biological compartments. For HPV-positive oropharyngeal cancers, plasma ctDNA typically offers higher diagnostic sensitivity than saliva due to rich lymphatic and vascular drainage [4]. Conversely, for HPV-negative oral cavity tumors, salivary sampling provides enrichment for locally shed biomarkers. Dual-fluid profiling, combining plasma for systemic burden and saliva for tumor-proximal signals, maximizes detection sensitivity across anatomical subsites [7].

The prognostic significance of liquid biopsy findings differs markedly by HPV status. In HPV-positive disease, ctHPV-DNA clearance during chemoradiotherapy is an early indicator of treatment response, often preceding radiographic changes by weeks. Persistent or rising post-treatment ctHPV-DNA predicts clinical recurrence with 4–6 months lead time over imaging, and undetectable post-treatment levels identify patients with exceptionally low relapse risk who are ideal candidates for de-escalation protocols. The negative predictive value of ctHPV-DNA surveillance exceeds 90% in large cohorts.

For HPV-negative HNSCC, MRD detection relies on tracking tumor-informed somatic mutations. Postoperative or post-therapeutic detection of such mutations is robustly associated with significantly shorter recurrence-free survival, with hazard ratios for overall survival exceeding 20 in some series. However, the absence of a universal viral marker necessitates tissue availability and more complex assay workflows, limiting widespread adoption (Table 3) [31].

## 7. Integrative and Multimodal Strategies

**Figure 4:**
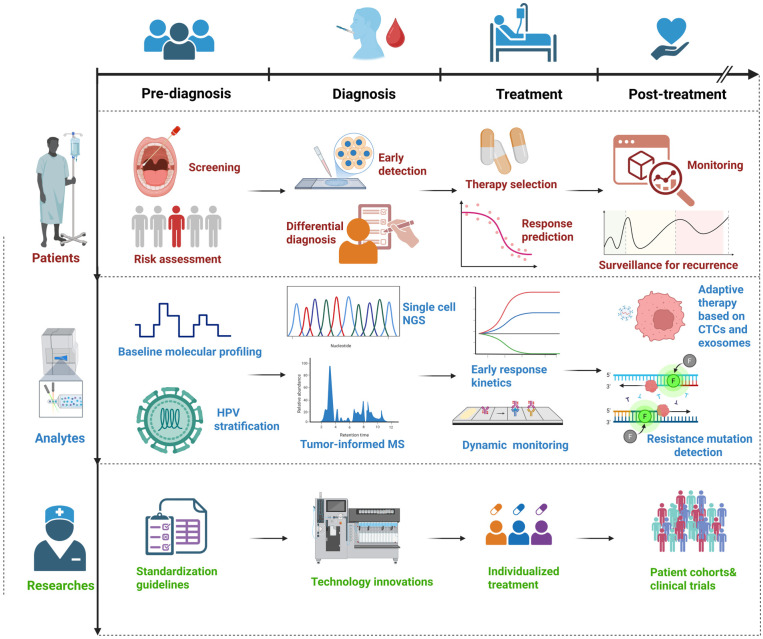
**Integrated roadmap for liquid biopsy translation in HNSCC.** The schematic illustrates the synergistic translation of liquid biopsy across the HNSCC treatment. The horizontal axis tracks the clinical journey from pre-diagnosis to post-treatment. Three aligned domains depict essential parallel developments: patients (evolving from screening to surveillance for recurrence), analytes (defining distinct viral to resistance mutation detection), and research (requiring standardization, innovation, and trials). This integrated framework highlights the coordinated advancements necessary to transition HNSCC management to a proactive, molecularly guided paradigm.

The three major liquid biopsy analytes, ctDNA, CTCs, and exosomes, differ fundamentally in their biophysical properties, analytical performance characteristics, and translational readiness. Understanding these quantitative differences is essential for selecting the appropriate tool for specific clinical applications and interpreting study results across platforms. However, it is important to acknowledge that fully integrated multi-analyte workflows have not yet been prospectively evaluated in HNSCC. The conceptual framework proposed below represents a synthesis of biological rationale and evidence from other tumor types, but prospective feasibility studies in HNSCC cohorts are urgently needed to validate whether combined analysis improves clinical outcomes beyond single-analyte approaches (Table 4 and Figure 4). ctDNA provides rapid, quantitative readouts of tumor burden. For example, plasma HPV16-ctDNA responses in oropharyngeal cancer often precede radiographic changes by weeks, underscoring its value as an early-warning MRD and surveillance tool [96,97]. PD-L1-positive exosomes track disease activity and predict response to immunotherapy [53,54], while dynamic changes in exosomal miRNA and protein cargo reflect ongoing tumor-immune interactions. CTCs, in turn, capture phenotypic plasticity and the emergence of resistant clones, with EPCAM/EGFR double-positive subsets associated with poor outcomes and indicative of evolving metastatic potential [98].

In clinical settings where resources are limited, a prioritization framework is necessary. The choice of which analyte to deploy should be guided by the specific clinical question and the biological characteristics of the tumor: (i) For MRD detection and recurrence surveillance in HPV-positive HNSCC, ctDNA (specifically ctHPV-DNA) should take precedence due to its high sensitivity, rapid turnaround time, and approaching clinical validation [73,74]. The clonal viral target provides an unambiguous tumor-specific signal that is technically straightforward to quantify. (ii) For HPV-negative HNSCC, where somatic mutations are heterogeneous and ctDNA assays require tumor-informed customization, ctDNA remains valuable but may be complemented by CTC enumeration when tissue is scarce for sequencing or when phenotypic information about EMT or metastatic potential is desired [61,75]. (iii) Exosomal PD-L1 profiling may take precedence. Studies showed that exosomal PD-L1 correlates with response to checkpoint inhibition and may outperform tissue PD-L1 immunohistochemistry [41,42] for assessment of tumor heterogeneity and clonal evolution, particularly at key decision points such as progression or treatment switch, CTC phenotyping provides unique single-cell resolution information that cannot be obtained from ctDNA alone [75]. When multi-analyte analysis is feasible, the combination should be tailored to the clinical scenario. For example, in a patient with HPV-positive oropharyngeal cancer completing curative-intent therapy, a rational approach would prioritize frequent ctHPV-DNA monitoring for early MRD detection, with exosomal immune profiling added if immunotherapy is being considered, and CTC analysis reserved for cases where metastatic progression or treatment resistance is suspected.

A practical clinical workflow could combine: (i) ctDNA assays for frequent, high-specificity surveillance; (ii) exosomal profiling for immune–tumor and viral monitoring, particularly in HPV-positive disease; and (iii) CTC phenotyping at key intervals to identify resistance or metastatic programs when tissue is scarce. Dual-fluid sampling strategies that test both saliva and plasma have demonstrated improved detection across anatomical subsites, particularly when HPV-associated biomarkers are included [4].

Large, prospective studies in other solid tumors illustrate the feasibility of AI-enabled cfDNA analytics at scale, though extrapolation to HNSCC MRD monitoring requires careful contextualization. The Circulating Cell-free Genome Atlas (CCGA) trial showed that machine-learning classifiers applied to targeted cfDNA methylation patterns can achieve very high specificity (99.5%) for multi-cancer early detection and with stage-dependent sensitivity (overall 51.5%; Stage I 16.8%, Stage II 40.4%, Stage III 77.0%, Stage IV 90.1%) and accurate cancer-signal origin prediction in close to 88.7% of cases [104]. However, the detection of incident cancers in asymptomatic individuals differs fundamentally from post-treatment MRD surveillance in HNSCC. In the MRD setting, the key clinical challenge is distinguishing true residual disease from treatment-related cfDNA background in patients with established cancer diagnoses. Therefore, we cite the CCGA study not as direct evidence for MRD monitoring, but rather as a proof-of-concept demonstrating the potential of this approach. Specifically, machine learning can integrate high-dimensional cfDNA features, such as methylation patterns and fragmentomic signatures, across large, multicenter cohorts, enabling algorithmic models to achieve the specificity thresholds required for potential clinical implementation. Applying similar strategies to HNSCC-specific MRD detection will require dedicated training cohorts composed of serially collected samples from uniformly treated patients with rigorously annotated clinical outcomes, as the underlying feature distributions (e.g., methylation patterns and fragment size profiles) are likely to differ between incident cancers and post-treatment MRD. Although fully integrated tri-analyte AI models combining ctDNA, exosome, and CTC data have not yet been applied in HNSCC, and significant feasibility barriers remain, evidence from other cancers suggests that such multimodal approaches could eventually facilitate personalized, dynamic disease monitoring and risk stratification once foundational work in assay standardization, cost reduction, and prospective validation has been completed. For now, these integrated strategies remain aspirational, and their clinical translation will require dedicated feasibility studies that address the practical constraints outlined above.

While the conceptual framework for integrated multi-analyte profiling is biologically compelling, its practical implementation in HNSCC faces substantial hurdles that must be transparently acknowledged. As summarized in Table 4, comprehensive analysis of ctDNA, exosomes, and CTCs would require approximately 25–35 mL of blood per time point (ctDNA: 15–20 mL; exosomes: >5 mL; CTCs: 5–10 mL) [104]. Such requirements may limit feasibility for longitudinal monitoring, particularly in elderly or heavily pretreated patients with HNSCC. The associated financial burden is also considerable, with research-grade tri-analyte profiling estimated to cost approximately $1300–$5800 per time point. In addition to cost and sample volume, turnaround time and logistical complexity represent important barriers. ctDNA analysis requires rapid sample processing, typically plasma separation within 2–4 h, or the use of specialized stabilization tubes due to its short half-life (~15 min to 2 h) and susceptibility to genomic DNA contamination from leukocyte lysis. Exosomes are more stable during freeze-thaw cycles because of their phospholipid bilayer; however, their isolation often relies on time-intensive procedures such as ultracentrifugation, which can require 6–24 h and delay result reporting. CTC analysis presents additional constraints, as immediate enrichment is typically necessary to preserve cell viability and prevent degradation. Coordinating these distinct technical workflows within a single clinical laboratory infrastructure remains technically challenging and resource-intensive. Analytical sensitivity and background noise further complicate interpretation. Although ctHPV-DNA assays can achieve exceptionally high analytical sensitivity (down to ~0.0025% mutant allele frequency) in HPV-positive disease [43], tumor-derived exosomes represent less than 1% of total circulating extracellular vesicles, and CTCs are detected in only 50–70% of patients, even when optimized detection platforms are used [82]. Consequently, a negative result from any single analyte cannot definitively exclude the presence of residual disease. Moreover, discordant findings across analytes (e.g., ctDNA-positive/CTC-negative) may create clinical management challenges in the absence of prospective data to guide their interpretation.

For most clinical scenarios, single-analyte strategies that focus on the most informative biomarker, such as ctHPV-DNA in HPV-positive HNSCC and tumor-informed ctDNA in HPV-negative HNSCC, offer the most practical balance of feasibility, cost-effectiveness, and clinical utility. Although multi-analyte profiling is conceptually appealing, its implementation should be selective and supported by a clear rationale. The most appropriate contexts for multi-analyte approaches include dedicated research studies aimed at understanding the biological complementarity among circulating analytes; specific clinical situations in which tumor tissue is unavailable for sequencing and phenotypic information is essential to guide management; and rigorously designed clinical trials evaluating whether combined analyte analysis provides meaningful improvements in clinical outcomes compared with single-analyte strategies.

## 8. Conclusions

Liquid biopsy is poised to reshape surveillance and therapeutic monitoring in HNSCC, yet its full potential remains contingent on a rigorous, evidence-based translation from the laboratory to the clinic. Among the triad of analytes, ctDNA has the highest level of clinical maturity potential [105]. In HPV-positive HNSCC, it stands as a validated prognostic biomarker with near-practice-changing potential, capable of detecting recurrence months before imaging. In HPV-negative HNSCC, tumor-informed ctDNA assays provide powerful prognostic information, though their complexity and cost currently limit widespread adoption. Exosomes and CTCs, in contrast, remain primarily within the domain of hypothesis-generating research. While they offer invaluable complementary insights into immune modulation, viral biology, and intratumoral heterogeneity, their translation is stalled by a lack of methodological standardization and the absence of large, prospective validation studies.

Realizing the clinical promise of these platforms will require a clearly ranked set of research priorities. Firstly, the field must prioritize rigorous analytical validation and harmonization of pre-analytical workflows, particularly for exosomes and CTCs, to ensure that findings are reproducible across laboratories and not merely artifacts of specific platforms. This requires concrete steps: for exosomes, adherence to MISEV2018 guidelines, development of reference materials, and multi-center ring trials to quantify inter-laboratory variability [106,107]; for CTCs, cross-platform calibration studies, harmonized positivity thresholds, and adoption of MIACTCS reporting standards [108]. Without this foundational work, data from different studies cannot be compared or combined, and the biological insights generated remain unverifiable. Once standardized assays are established, large-scale, multicenter prospective cohort studies are needed to confirm the prognostic utility of these analytes in well-annotated HNSCC populations. Finally, interventional trials that use liquid biopsy results to guide treatment decisions, escalating or de-escalating therapy based on ctDNA MRD status, for example, can only be ethically and methodologically justified after analytical and clinical validity have been firmly established. This sequential prioritization ensures that the field advances from biological discovery to clinical impact in a rigorous, evidence-based manner. By integrating multimodal datasets within advanced, AI-enabled frameworks, and by anchoring these analyses in the specific biological context of HPV status and anatomic site, liquid biopsy can progress from an investigational modality to a central component of personalized, dynamic HNSCC care. However, achieving equitable implementation will require proactive efforts to address barriers to global access, including the high cost of NGS-based assays, limited laboratory infrastructure in low-resource settings, lack of regulatory harmonization across jurisdictions, and the need for affordable, point-of-care diagnostic platforms that can be deployed in diverse healthcare environments.

## Figures and Tables

**Table 1:** Summary of key studies investigating ctDNA as a biomarker in HNSCC.

Research Study	Detection	Key Findings	Limitations
HNSCC treated with radical therapy [20]	Tumor-informed plasma NGS	ctDNA positivity associated with significantly shorter relapse-free survival (9.6 vs. 20.6 months, *p* < 0.01).	Very small cohort (*n* = 20); retrospective analysis; single-center; lack of external validation; *p*-value presented without correction for multiple testing.
Non-metastatic HPV-positive OPSCC after curative chemoradiotherapy [21]	Plasma circulating tumor HPV DNA (ctHPV-DNA)	ctHPV-DNA enabled earlier salvage evaluation.	Observational design (*n* = 115); no control arm; single-center; lead time benefit quantified but impact on overall survival not yet demonstrated.
HPV-negative HNSCC treated with curative surgery [22]	Tumor-informed NGS (RaDaR) and serial plasma	Rising ctDNA predicted relapse in all progressing patients.	Very small, highly selected cohort (*n* = 17); tumor-informed approach is resource-intensive and not feasible for all patients; requires high-quality archival tissue.
HPV-positive OPSCC during post-treatment surveillance [23]	Tissue-modified viral HPV DNA assay (TTMV-HPV DNA)	80/1076 (7.4%) tested positive; 93% of initially unrecognized positives later confirmed as recurrence. PPV 95%, NPV 95% in real-world surveillance.	Large real-world cohort (*n* = 1076) is a strength, but PPV/NPV are prevalence-dependent; wide confidence intervals not reported; assay is specific to HPV16, excluding other high-risk HPV types.
HNSCC receiving immunotherapy (SHIZUKU-HN) [24]	Guardant360 NGS	VAF correlated with tumor volume (ρ = 0.70); ctDNA changes preceded radiographic progression; detected resistance mutations missed in tissue.	Small cohort (*n* = 27); observational design; C-CAT database analysis (*n* = 2255) shows only 7% testing rate, highlighting implementation gap.
R/M HNSCC receiving ICB [10]	Tumor-informed RaDaR	ctDNA negativity during treatment associated with 3-yr OS (HR 0.04) and disease control (OR 21.7).	Very small cohort (*n* = 16); impressive effect sizes but wide confidence intervals; requires validation in larger multicenter study.
Locally advanced HNSCC post-surgery [25]	Tumor-informed NGS	81.3% preoperative detection; one patient had 6.5 months lead time over imaging.	Preliminary study (*n* = 16); very small cohort; single case with dramatic lead time; requires confirmation.
HPV-independent HNSCC postoperative lymph ctDNA [26]	Tumor-informed sequencing of surgical drain fluid across two cohorts	Lymph ctDNA at 24 h predicted recurrence (sensitivity 76%/65%); matched plasma non-predictive (sensitivity 35%); intermediate-risk subgroup: 88% sensitivity, HR 8.4; all radiotherapy-only relapsers were lymph ctDNA-positive.	Two independent cohorts (*n* = 73) with replication; multi-site enrollment strengthens generalizability; intermediate-risk findings have immediate clinical relevance; requires prospective interventional validation.

**Table 2:** Summary of key studies investigating exosomes as a biomarker in HNSCC.

Research Study	Comparison with Imaging/Clinical Assessment	Key Qualitative Findings	Limitations
HNSCC patients; plasma-derived EVs [53]	PD-L1^+^ exosome counts declined to baseline within 4 weeks post-therapy, preceding imaging normalization by ≈2 months	Exosomal PD-L1 tracked tumor burden and immunosuppression; potential early relapse indicator	Small cohort (*n* = 40); lead time estimated but not rigorously quantified; lack of a standardized cut-off for “positivity”; functional immune assays not linked to clinical outcomes.
HNSCC patients and healthy donors; plasma EVs [54]	No lead time reported	Plasma EVs carried immunosuppressive cargo and inhibited lymphocyte function; tracked clinical disease activity	Small cohort (*n* = 38 HNSCC patients and *n* = 14 healthy donors); in vitro functional data may not fully represent in vivo complexity; cross-sectional design limits ability to assess dynamic tracking.
OPSCC; saliva-derived EVs [55]	Detected exosomal HPV DNA in 80% of pathologically confirmed cases	Rapid, label-free salivary exosome assay accurately identified HPV-positive OPC.	Very small, proof-of-concept study (*n* = 10); no blinding; highly selected population; technology not widely available; specificity not assessed in a cohort with benign oral disease.
OSCC cases and healthy saliva controls; matched tumor tissue samples [56]	Elevated miR-24-3p distinguished OSCC from controls when oral exam was nonspecific	miR-24-3p promotes OSCC proliferation; shows promise as an early detection biomarker	Small sample size (*n* = 49 OSCC patients and *n* = 14 healthy donors); AUC of 0.738 is modest for a diagnostic test; no independent validation cohort; lacks specificity against other oral inflammatory conditions.
Saliva ± matched tumor/cell lines [57]	EV miRNA alterations detectable before biopsy; differentiated tumor vs. healthy	EV miRNA signatures associated with EMT and nodal metastasis; supports multi-miRNA panel use	Discovery-phase study; unclear if findings are reproducible across different isolation platforms; no prospective validation in a screening setting.
HNSCC patients; saliva-derived exosomes [58]	No lead time reported	Saliva richer in tumor-derived exosomes than plasma; exosomes attenuated CD8^+^ T cell activity and produced adenosine	Moderate cohort size (*n* = 21); comprehensive multi-parametric analysis; functional validation strengthens findings; single-center; requires external validation in larger cohort.
HNSCC patients (n = 11); paired plasma/saliva exosomes [59]	No imaging comparison	119 miRNAs overlapped between biofluids; 29 tumor-exclusive miRNAs identified	Small cohort (n = 11 patients + 5 HD); paired biofluid design is a strength; discovery-phase findings require validation; correlation with DFS preliminary.
HNSCC patients (n = 25); plasma-derived CD16+ exosomes [60]	No imaging comparison	CD16+ exosomes correlate with CD16+ monocyte subsets and adhesion molecules (CD29, CX3CR1)	Correlative findings; mechanistic link between exosomal CD16 and monocyte modulation requires further study; sample size not specified.

**Table 3:** Biological and analytical differences in liquid biopsy analytes between HPV-positive and HPV-negative HNSCC.

Feature	HPV-Positive HNSCC	HPV-Negative HNSCC
ctDNA		
Primary biomarker	Clonal HPV16 E6/E7 DNA	Somatic mutations (e.g., TP53, NOTCH1, CDKN2A)
Optimal biofluid	Saliva (higher concentration for oropharyngeal) and plasma	Primarily plasma
Detection method	Ultra-sensitive ddPCR	Tumor-informed sequencing or NSG
Clinical utility	High negative predictive value for recurrence	Requires tumor-informed approach for MRD
Exosomes		
Cargo	Viral (E6/E7 DNA/RNA/proteins) and host miRNAs	Host-derived related to inflammation and EMT
Immune modulation	May have distinct immune interactions	Often more immunosuppressive
Clinical utility	Diagnostic via viral cargo	Prognostic biomarkers
CTCs		
Phenotype	EMT/partial-EMT, low EpCAM	High EMT, higher EpCAM and EGFR
Capture method	Label-free (size, density) essential	EpCAM-based methods (e.g., CellSearch) and label-free
Clinical utility	Viral transcripts	Enumeration and phenotypic characterization
MRD Detection Sensitivity	Very high for ctHPV-DNA	Moderate to high with tumor-informed assays

**Table 4:** Quantitative comparison of liquid biopsy analytes.

Parameter	ctDNA	Exosomes	CTCs
Analytical Sensitivity	0.0025% mutant allele frequency (CAPP-Seq with error suppression) [44] 25 aM (electrochemical detection) [99] 0.3 aM-0.1 fM (nanotechnology platforms) [100]	32–100 exosomes/μL (nano technology platforms) 9.08 × 10^3^ particles/μL (MOFs ECL assay) 37.5 exosomes/mL (polymer nanoparticles) [99]	1–10 cells/mL (typical range) 1 cell/mL (electrochemical detection) 0.25 cells/mL (fluorescence detection) 2 CTCs/4 mL (graphene oxide) [100]
Clinical Sensitivity (HNSCC)	HPV-positive: 89% pretreatment detection [101]; ~95% in metastatic HPV-negative: 77–81% in primary tumor [25,30,101]; 50–70% in early-stage	Not well quantified due to dilution by normal cell EVs Estimated tumor-derived EV fraction: <1% of total EVs [102]	50–70% detection rate across stages 51.2% (p16+ CTCs) 70.7% (p16- CTCs) [82]
Clinical Specificity	Very high (>95%) for mutation-specific assays HPV-DNA assays: 94–99% [102]	Moderate (75–90%) Challenging to distinguish tumor-derived from host EVs [102]	High (68–90%) EpCAM-based selection with CD45 exclusion minimizes false positives [102]
Sample volume required	15–20 mL blood/plasma Saliva: 2–5 mL [103]	>5 mL blood Saliva: 2–5 mL [103]	5–10 mL blood [103]
Background noise	Moderate (normal cfDNA interference) ctDNA fraction as low as 0.01% in early-stage	Moderate–High (interference from non-tumor EVs, lipoproteins, protein aggregates)	Low (intact cells with positive selection)
Turnaround time	3–7 days (NGS-based) 4–6 h (ddPCR) <1 h (prototype POCT)	6–24 h (ultracentrifugation-based) 2–4 h (bead-based capture + proteomics)	4–8 h (enrichment + staining + enumeration) Dependent on isolation method
Cost	High: $500–$3000 (NGS) Moderate: $100–$300 (ddPCR)	High: $300–$1000 (ultracentrifugation + MS) Moderate: $100–$300 (bead-based kits)	High: $500–$1500 (CellSearch^®^) Moderate: $200–$600 (research platforms)
Repeatability	Moderate–high: standardized NGS/ddPCR workflows; inter-laboratory variation exists	Low: pronounced methodological heterogeneity	Low-moderate: highly platform-dependent; low repeatability noted
Stability	Low: short half-life (~15 min-2 h); requires rapid processing or stabilization tubes	High: phospholipid bilayer protects cargo; stable through freeze-thaw	Low: require prompt processing; viable CTCs degrade within 24–48 h
Technical difficulty	Moderate: standardized extraction and NGS/ddPCR workflows	Very difficult: Isolation technically challenging; no standardized protocols	Difficult: complex enrichment and identification
Information loading	Genetic: mutations, methylation, fragmentomics, copy number variations	Multi-omics: proteins, RNAs (miRNA, mRNA, lncRNA), lipids, DNA	Comprehensive: genome, transcriptome, proteome, epigenome at single-cell level
Heterogeneity representation	Low-moderate: average signal from multiple tumor clones; can detect subclonal mutations if >0.1% AF	Moderate: bulk EV population reflects average; single-EV analysis emerging	High: single-cell resolution enables clonal architecture mapping; but may miss some clones due to low capture
Difficulty of standardization	Moderate: multiple platforms but emerging proficiency testing	High: extreme methodological heterogeneity; MISEV guidelines emphasize transparent reporting	High: no harmonized protocols
Regulatory status (HNSCC)	None approved specifically for HNSCC Multiple assays approved for other cancers. Guardant360^®^ (Palo Alto, CA, USA), FoundationOne^®^ (Boston, MA, USA), Signatera™ (San Carlos, CA, USA).	None approved	CellSearch^®^ (Huntingdon Valley, PA, USA) FDA-cleared for metastatic breast, prostate, colorectal cancer; not approved for HNSCC
Primary clinical applications (Current)	MRD detection (research) Therapy response monitoring Early relapse detection Genomic profiling	Early detection research Immune monitoring (PD-L1+ EVs) Intercellular communication studies Potential drug delivery vehicles	Prognostic stratification Pharmacodynamic biomarker Functional studies (cultures, xenografts)
Translational readiness (HNSCC)	High (rapidly advancing): validated prognostic utility; entering guidelines for other cancers; HNSCC validation ongoing	Low–intermediate: preclinical/early clinical; biologically promising but clinically premature	Intermediate: prognostic signal established; detection rates and platform heterogeneity limit clinical use

## Data Availability

No new data were created or analyzed in this study. Data sharing is not applicable to this article.

## Abbreviations

AUC	Area under the curve
AML	Acute myeloid leukemia
CAPP-Seq	Cancer Personalized Profiling by Deep Sequencing
CCGA	Circulating Cell-free Genome Atlas
CfDNA	Cell-free DNA
CHIP	Clonal hematopoiesis of indeterminate potential
CtDNA	Circulating tumor DNA
CT	Computed tomography
CTCs	Circulating tumor cells
DC	Dendritic cell
DEASH	Differential enrichment of allele-specific hybrids
E6/E7	HPV oncogenes E6 and E7
EMT	Epithelial–mesenchymal transition
EpCAM	Epithelial cell adhesion molecule
EVs	Extracellular vesicles
HNSCC	Head and neck squamous cell carcinoma
HPV	Human papillomavirus
HPV16	Human papillomavirus type 16
HR	Hazard ratio
LA-HNSCC	Locally advanced head and neck squamous cell carcinoma
MRD	Minimal residual disease
miRNA/miR	MicroRNA
MRI	Magnetic resonance imaging
NaME-PrO	Nuclease-assisted minor-allele enrichment with probe overlap
NGS	Next-generation sequencing
OSCC	Oral squamous cell carcinoma
PET	Positron emission tomography
PBMC	Peripheral blood mononuclear cell
VAF	Variant allele fraction
